# Stakeholders’ Views on Information Needed in a Patient Decision Aid for Microtia Reconstruction

**DOI:** 10.1177/10556656221146584

**Published:** 2023-01-05

**Authors:** E.M. Ronde, Veronique A.P. van de Lücht, N. Lachkar, Dirk T. Ubbink, Corstiaan C. Breugem

**Affiliations:** 1 Department of Plastic, Reconstructive and Hand Surgery, Amsterdam UMC location University of Amsterdam, Amsterdam, the Netherlands; 2 Department of Surgery, Amsterdam UMC location University of Amsterdam, Amsterdam, the Netherlands; 3 Amsterdam Reproduction and Development, Amsterdam, the Netherlands; 4 Personalized Medicine, Amsterdam Public Health, Amsterdam, the Netherlands

**Keywords:** microtia, ear reconstruction, shared decision-making, patient decision aid, qualitative research, PADAMIR

## Abstract

**Objective:**

To assess which information about microtia and the possible reconstructive options health care providers (HCPs), patients and parents believe should be included in a patient decision aid (PtDA).

**Design:**

A mixed-methods study comprised of an online survey of HCPs and focus group discussions with patients and parents.

**Participants:**

Survey respondents were members of the International Society for Auricular Reconstruction (ISAR). Focus group participants were patients with microtia and their parents, recruited through the microtia outpatient clinic at Amsterdam UMC, and through a Dutch patient organization for cleft and craniofacial conditions.

**Methods:**

An online, investigator-made survey was sent to ISAR members in December 2021. Semi-structured focus group discussions were held in February 2022. Quantitative results were summarized, and qualitative results were thematically grouped.

**Results:**

Thirty-two HCPs responded to the survey (response rate 41%). Most respondents (n = 24) were plastic surgeons, who had a median of 15 years of experience (IQR: 7-23 years). Two focus groups were held with a total of five patients and two parents. HCPs, patients and parents generally agreed on the information needed in a PtDA, emphasizing the importance of realistic expectation management. Patients and parents also considered psychosocial and functional outcomes, patient experiences, as well as patients’ involvement in decision-making important.

**Conclusions:**

A PtDA for microtia reconstruction should target all patients with microtia, and include information on at least technique-related information, expected esthetic results, possible adverse effects, psychosocial and functional outcomes and patient experiences. Preference eliciting questions should be developed for both pediatric patients and their parents.

## Introduction

Microtia is a congenital condition characterized by hypoplasia or aplasia of the external ear and ear canal.^1^ It can occur with or without apparent asymmetry of the face, a widely accepted feature of craniofacial microsomia,^2^ where some even consider microtia a mild form of this condition.^3^

Ear reconstruction may be considered due to esthetic, functional or psychosocial reasons^4^ and is traditionally performed using an implant shaped from autologous costal cartilage (ACC).^5,6^ Alternatively, alloplastic porous polyethylene implants (PPE) and osseointegrated (OIP) or adhesive-retained (ARP) auricular prostheses may provide good esthetic results without rib donor site morbidity.^7,8^ Furthermore, patients may opt to not undergo ear reconstruction or prosthetic placement.^9,10^ As all of these treatment options are accompanied by various advantages and disadvantages,^11,12^ patients’ and their parents’ preferences should play a vital role during decision-making, so that the chosen treatment best suits these preferences. The bidirectional communication between a health care provider (HCP) and a patient and their parents that facilitates this, is known as shared decision-making (SDM). This communication should lead to a treatment-related decision based on evidence-based information about the treatment options, including their advantages and disadvantages, as well as the patient's values and preferences.^13^ SDM seems like a valuable communication approach, considering that there is convincing evidence that applying SDM improves patient knowledge and active patient involvement.^13^

Patient decision aids (PtDAs) are tools that facilitate SDM by improving patients’ knowledge of treatment options and reducing decisional conflict.^14,15^ A PtDA typically contains information about the condition, up-to-date literature evidence on the benefits and possible harms of the treatment options, as well as questions that help patients explore their personal preferences.^16^ A previous study found that parents of patients with microtia consider receiving information about the treatment options a very important part of counseling.^17^ A PtDA will not only provide reliable information about the treatment options, it will also encourage patients and their parents to participate in the decision-making process.

This study aimed to summarize which information patients with microtia, their parents, and HCPs who treat patients with microtia consider important to include in a PtDA for microtia reconstruction. This study is a part of the larger PADAMIR-project (PAtient Decision Aid for MIcrotia Reconstruction) aiming to develop a PtDA for microtia reconstruction.

## Methods

The Medical Ethics Review Committee at Amsterdam UMC waived the need for a full ethics review of the PADAMIR project (W21_366 # 21.407). The Checklist for Reporting Of Survey Studies (CROSS)^18^ (Appendix A), and the Consolidated criteria for reporting qualitative research (COREQ)^19^ (Appendix B) checklists were followed for the survey and focus groups, respectively.

### Survey Methodology

HCPs’ views were collected through an online cross-sectional survey, using Castor Electronic Data Capture.^20^ The survey questions were developed by the first and senior authors and consisted of a maximum of 22 questions: six questions on participant characteristics and 16 questions on PtDA content (Appendix C). Questions on the advantages and disadvantages of individual treatment options were only displayed (Questions 3.2-3.7, Appendix C) if the specific treatment option was checked in the previous question (question 3, Appendix C).

The survey was distributed using purposive sampling: a survey invitation was sent to all members of the International Society of Auricular Reconstruction (ISAR) using the society's mailing list in December 2021, and the link to the survey was shared in the WhatsApp group for members. A reminder was sent via WhatsApp in February 2022. ISAR members were approached for this study as it is a society for HCPs involved in clinical ear reconstruction care and research, and all members must have at least three years of experience in ear reconstruction before joining. Repeated participation was controlled by checking for identical participant characteristics in the summarized survey results. Answers were collected anonymously, unless participants chose to share their contact details to indicate they wished to participate in future PtDA prototype testing. If so, contact details were separated from survey responses before data analysis to ensure confidentiality.

### Focus Group Methodology

Patients and parents’ views were obtained through two online focus groups that were conducted according to established methodology in February 2022.^21^ Patients with microtia were eligible to participate if they had undergone ear reconstruction or ear prosthesis placement or had consciously elected not to undergo such an intervention. Parents of these patients were also encouraged to join. Thirty eligible patients treated at the microtia outpatient clinic at Amsterdam UMC were randomly selected and invited to participate by e-mail. Participants were also recruited through an online presentation at the members’ meeting of Laposa, a Dutch patient organization for cleft palate and craniofacial conditions. Furthermore, information about the project and contact details for participation were placed on the organization and our hospital's websites.

An experienced moderator (DTU) and two observers (EMR, VAPL) led the focus groups. A guide with overarching subject matters and supporting questions was developed in advance by the authors (Appendix D). The focus groups were audio-recorded (with participant consent), and field notes were made on any relevant non-verbal communication. Participants were also given the opportunity to contact the researchers to share any additional views after the focus group.

### Data Analyses

Quantitative data were analyzed using SPSS (v26.0, IBM Corp, Armonk, NY, USA),^22^ and are presented as numbers (N) and proportions (%) of (survey) respondents, or medians with interquartile ranges, where applicable. Questions were summarized individually, and the numbers of respondents per question are shown in the relevant tables. Consensus was defined as ≥75% agreement on an answer option in the HCP survey. Qualitative survey answers were coded by one author (EMR) and displayed visually in a figure or a code tree. One author (VAPL)transcribed the focus group recordings and supplemented these with field notes. Two authors (EMR, VAPL) subsequently coded the transcripts; the authors first defined initial deductive codes together based on the focus group discussions and a general read-through of the transcripts. The same authors then coded the transcripts independently. Consensus on the assigned codes was reached through discussion, and codes were slightly amended where needed. Themes were derived from the codes through collaborative discussion and were displayed in a code tree.

## Results

### Survey

Thirty-two ISAR members responded to the survey (41% response rate). Most of the respondents (75%) were plastic surgeons, and 63% worked in a university hospital. They had a median of 15 (IQR 7-23) years of experience. Respondents were most frequently from Europe, Asia, or North America, and most of them (88%) performed at least ACC reconstructions (Table 1).

**Table 1.** Survey Respondent Characteristics (n = 32).

Characteristic	Number of respondents (%)
Discipline	
Plastic surgeon	24 (75)
ENT surgeon	6 (19)
Other^a^	2 (6)
Setting	
University hospital	20 (63)
Private clinic	11 (34)
Other^b^	1 (3)
Years of experience	Median 15; IQR (7-23)
Type of treatment performed	
ACC reconstruction	28 (88)
PPE reconstruction	13 (41)
ARP placement	10 (31)
OIP placement	6 (19)
Other^c^	1 (3)
Location	
Europe	12 (38)
Asia	8 (25)
North America	7 (22)
South America	3 (9)
Oceania	2 (6)

Abbreviations: ENT, Ear-, nose- and throat surgeon (otorhinolaryngologist); ACC, autologous costal cartilage; PPE, porous polyethylene; ARP, adhesive-retained prosthesis; OIP, osseointegrated prosthesis.

^a^ Other disciplines: specialist nurse, clinical psychologist.

^b^ Other setting: private and hospital.

^c^ Other treatment: none.

**Figure 1.**
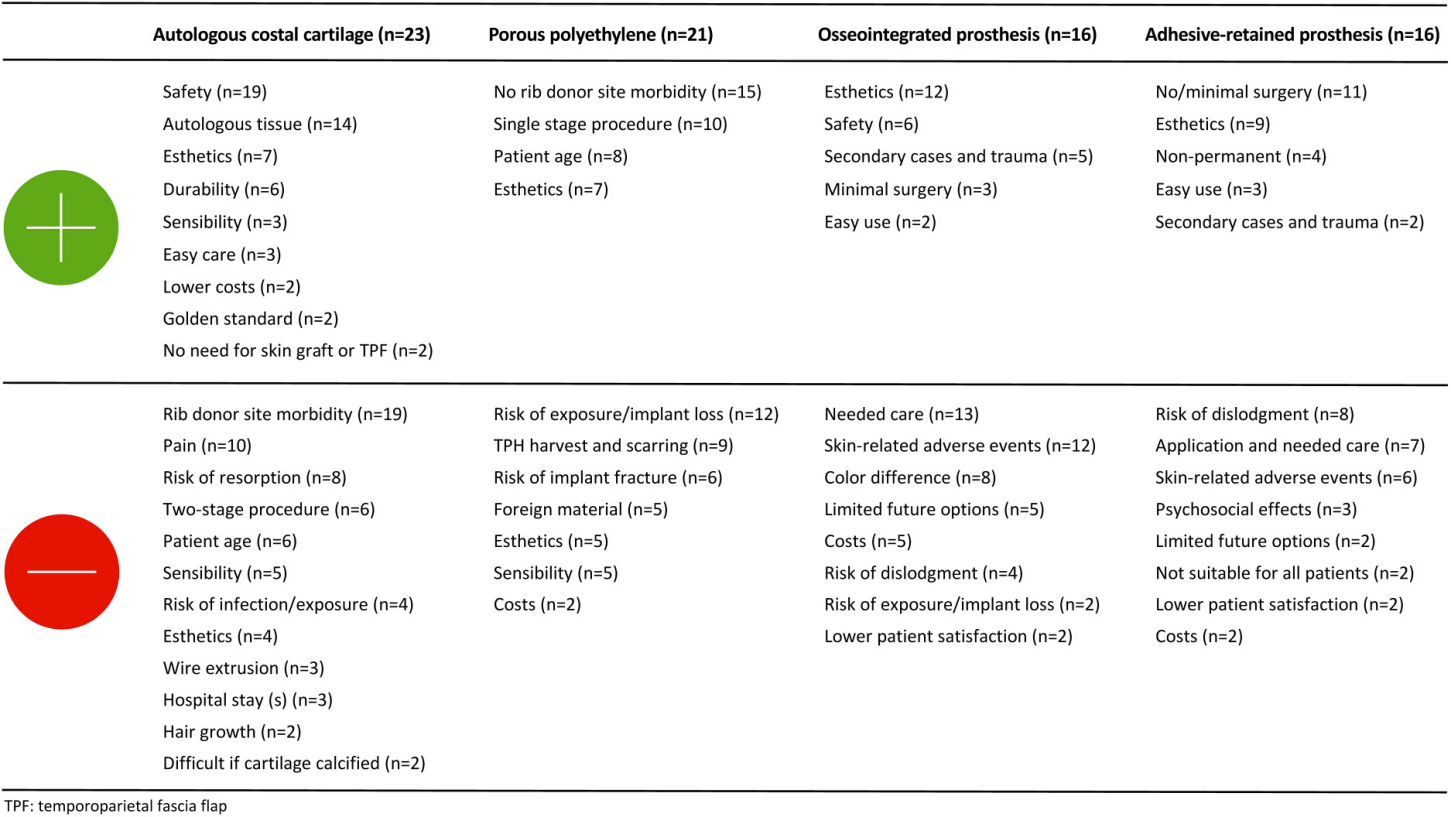
Advantages and disadvantages of autologous costal cartilage reconstruction, porous polyethylene implant reconstruction, osseointegrated prostheses and adhesive-retained prostheses according to health care providers.

HCPs agreed that a PtDA on microtia reconstruction should contain information about ACC (100%) and PPE reconstruction (90%), as well as general information on hearing, associated conditions, and the psychosocial effects of having microtia. Regarding the treatment options, respondents agreed on including information related to the procedure, the expected esthetic results, postoperative pain, and adverse events (Table 2). The most frequently named advantages and disadvantages of ACC, PPE, OIP and ARP are shown in Figure 1.

**Table 2.** Health Care Providers’ Answers to Checkbox Questions.

Question and answer options	Proportion of respondents (%)
Information on microtia that should be included (n = 32)	
Types of microtia	22 (69)
Incidence of microtia	17 (53)
Male to female ratio	6 (19)
Heredity	20 (63)
Associated syndromes and conditions	25 (78)
Psychosocial effect	24 (75)
Hearing	29 (91)
Reconstruction techniques that should be included (n = 30)	
ACC	30 (100)
PPE	27 (90)
OIP	22 (73)
ARP	21 (70)
Info on outcomes that should be included (n = 24)	
Age	24 (100)
Expected aesthetic result	24 (100)
Autologous vs alloplastic materials	23 (96)
Length of hospital stay	23 (96)
Consequences for atresiaplasty or BAHA	79 (92)
Length of surgeries	22 (92)
Number of surgeries	22 (92)
Risk of adverse events	22 (92)
Types of adverse events	22 (92)
General information on operation (technique)	21 (88)
Expected longevity of aesthetic result	20 (83)
Expected amount of postoperative pain	20 (83)
Number of incisional scars	20 (82)
Expected auricle growth	17 (71)
Expected sensibility of auricle	17 (71)
Data on PROMS regarding functional effects	14 (58)
Data on PROMS regarding psychosocial health	13 (54)
Data on PROMS regarding emotional health	13 (54)
Detailed (step-by-step) information on operation technique	13 (54)
Media that should be used (n = 24)	
Pictures	23 (96)
Illustrations	19 (79)
Video	15 (63)
Patient stories	14 (58)
Text	13 (54)
Animation	11 (46)
Other: website	1 (4)

Abbreviations: ACC, autologous costal cartilage reconstruction; PPE, porous polyethylene reconstruction; OIP, osseointegrated prosthesis; ARP, adhesive-retained prosthesis; BAHA, bone-anchored hearing aid; PROMS, patient-reported outcome measures.

Twenty-eight survey respondents (88%) provided one or more qualitative comment(s). Themes derived from the qualitative comments were (1) information about treatment options, (2) psychosocial support, (3) factors that influence decision-making and (4) options for hearing (Supplementary Figure 1). Seven (22%) respondents commented on the importance of providing realistic information about the expected esthetic results and discussing the option of not undergoing reconstructive treatment. For example, one respondent commented that “standardized photos, including all angles of patients before and after, [should be provided] so a family can have a realistic idea what they can expect for their child with each surgeon they are considering.” Four respondents (13%) noted that providing psychological support in the form of education or counseling is important, where one respondent stated that “with the right education and support encouraging resilience and self-acceptance, reconstructive surgery should only be a plan B.”

### Focus Groups

Eleven individuals expressed interest in participation, of whom eight consented to participate, and seven attended the online focus groups. Six of these participants were recruited through the outpatient clinic, and one participant referred themselves after attending the online presentation at the Laposa meeting. Four participants attended the first, and three attended the second focus group. Five participants were patients, with a median age of 18 (IQR 18-53) years. Most patients had undergone ACC reconstruction, and the patients’ median age at the time of surgery was 11 (IQR 9.5-13) years (Table 3).

**Table 3.** Focus Group Participant Characteristics (n = 7).

Characteristic	Number of participants
Patients	5
Males	4
Treatment	
ACC reconstruction	4
PPE reconstruction	1
N/A^a^	2
Diagnosis	
Isolated microtia	3
Craniofacial microsomia	2
N/A^a^	2
Age, all participants	Median 20, IQR (18-53)
Age, patients only	Median 18, IQR (15-30)
Age at primary surgery, patients only	Median 11, IQR (9.5-13)
Highest level of education	
Primary school	1
Secondary school	4
Higher vocational education	1
University	1

Abbreviations: ACC, autologous costal cartilage; PPE, porous polyethylene; N/A, not applicable.

a Participants were parents whose children had undergone ACC reconstruction (parent 1) and PPE reconstruction (parent 2), and were diagnosed with isolated microtia.

**Figure 2.**
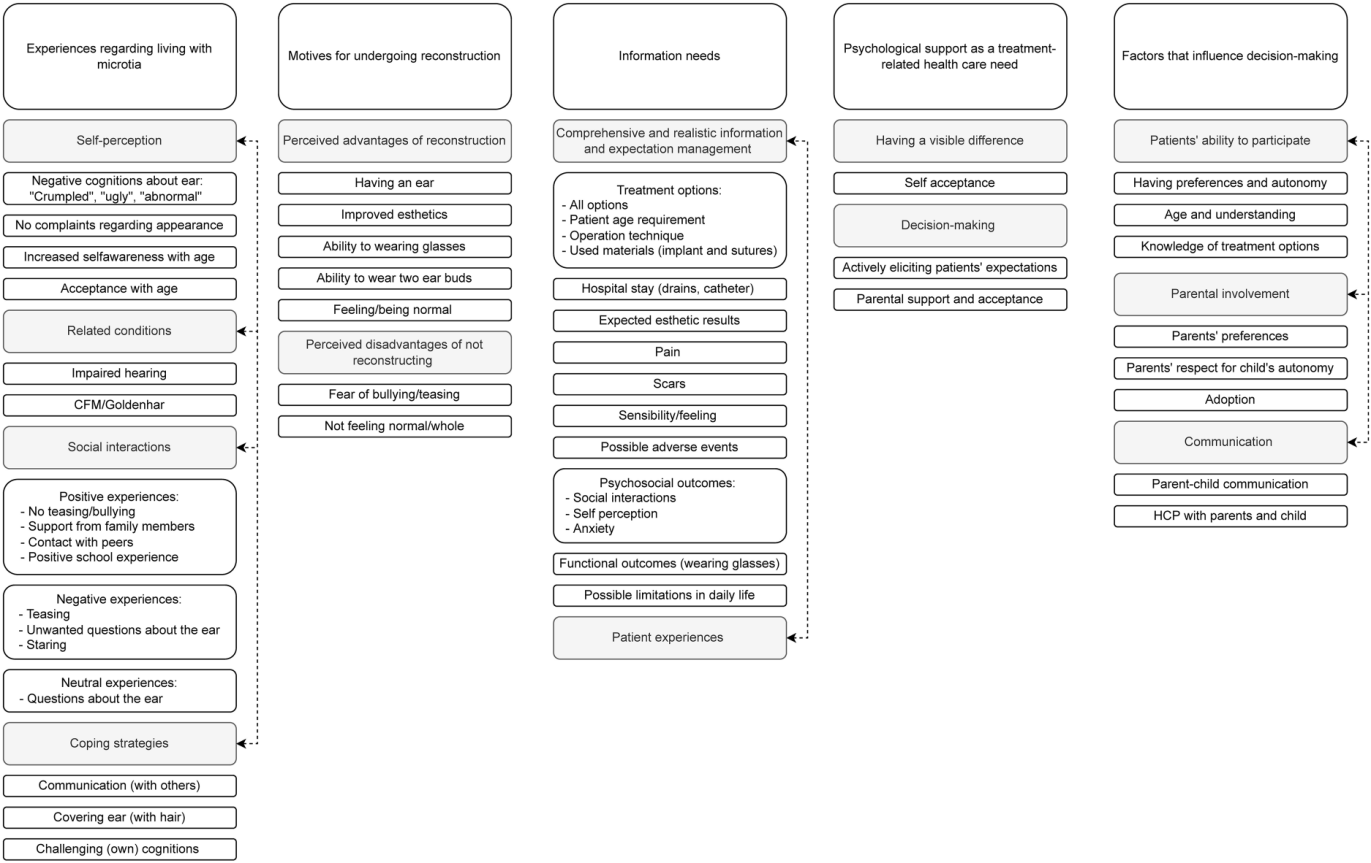
Code tree of themes derived from focus group discussions. Related subthemes are connected by dashed lines.

Identified themes were (1) experiences regarding living with microtia, (2) motives for undergoing reconstruction (3) information needs, (4) psychological support as a treatment-related health care need and (5) factors influencing decision-making (Figure 2).

#### 1. Experiences Regarding Living with Microtia

According to focus group participants, their or their children's experiences regarding living with microtia were shaped by their self-perception, varying social interactions, coping strategies, as well as related conditions, such as craniofacial microsomia.

When recalling their experiences before treatment, most participants described their ear as “ugly”, “crumpled” and “something that can't be called an ear” or expressed negative cognitions about themselves like “not feeling complete enough” or not being “normal”. One participant mentioned that they were not bothered by their ear due to their more conspicuous features related to craniofacial microsomia. Most participants had experienced negative social interactions, such as unwanted questions about their ear and staring:

* “I noticed that [the teasing] gradually started when I was 10 years old: people asking about [the affected ear], staring at [me], or having something to say about [the ear], unfortunately.”*

However, despite this, several participants explicitly stated that they had not been teased or bullied. Furthermore, various coping strategies, including covering the affected ear with hair, communicating with others about the condition and challenging their own cognitions, as well as positive social interactions and peer support were important facilitators of a positive experience:

*“I’m a member of the patient organization Laposa, so I know people through the organization now. It*‘*s nice to know that there are other people like me. They understand the bit about looking different and being stared at. It*‘*s nice to meet like-minded people.”*

#### 2. Motives for Undergoing Reconstruction

All participating patients had undergone ear reconstruction. Motives named for choosing surgery were esthetic and functional considerations, negative social interactions, as well as psychosocial reasons like wanting to be normal or a fear of bullying.

Specific motives for opting for ACC were esthetics (n = 2) and having a real ear (n = 1). For PPE these were not having to harvest cartilage (n = 1) and surgery being possible at a younger age (n = 1).

#### 3. Information Needs

##### Subtheme Comprehensive and Realistic Information, and Expectation Management

There was an overall consensus on the need for comprehensive and realistic information regarding the treatment options, hospital stay, expected outcomes, as well as possible adverse events. Information that was considered important included clear explanations of the procedure, the types of materials used and age requirements. Participants also considered discussing the option of not undergoing surgery important. Six out of the seven participants did not regret having had reconstructive surgery or that their child had had surgery.

*“I haven*‘*t experienced any lasting problems, and I am very grateful that [ear reconstruction] was possible, because if this had not happened, what would [my life] have been like? Would people have stared at [me]? Because I think that life would have been very different if I hadn*‘*t [had my ear reconstructed]. And that*‘*s why I would do it 100 times over now that I know what you get in return.”*

However, all participants described unexpected treatment outcomes and agreed on the importance of good expectation management:

*“When I saw [the ear] for the first time, I was really disappointed. It was really swollen, and I thought: if this is it, just go ahead and remove it. But slowly, I really had to be patient for a long time, the swelling went down.”*

*“I expected [the reconstructed ear] to be soft, but it*‘*s really hard, and I think I would have liked to have known that. It*‘*s also still pretty thick. I thought that it would have been thinner.”*

*“[The information in a PtDA] shouldn*‘*t be too [optimistic]. So not like ‘the results are super good and surgery isn*‘*t painful’. I think it should be made really clear that [ear reconstruction] is an intense procedure; the rib cartilage procedure, in particular.”*

Participants also suggested including pictures from all angles of the ear, as well as at various postoperative stages.

Furthermore, all participants experienced adverse events after surgery. The most frequently mentioned adverse events were hair growth on the ear (n = 3), infection (n = 2), alopecia (n = 2), postoperative nausea (n = 2) and wire protrusion (n = 1). Effects of the operation on psychosocial and functional outcomes were also discussed. Participants shared experiences varying from not having any complaints in their daily lives to persistent negative social interactions and lingering anxiety after surgery due to pain.

*“I still prefer to hide [the ear] actually. Because people still look at it as it*‘*s still not a normal ear.”*

*“People don*‘*t always notice [my ear] anymore or anything. But most of the time I just tell people I’m deaf because that*‘*s easier for them to know…But sometimes, I don*‘*t tell people until after I’ve already worked with them for three months, and then they’ll be like ‘wow! I’ve never [noticed] that before’. And I like that as well: that people don*‘*t always notice it right away.”*

##### Subtheme Patient Experiences

Participants in both focus groups suggested adding patient experiences to the PtDA, to underscore possible psychosocial outcomes and postoperative quality of life.

* “The motives for undergoing reconstruction, the details that you miss in the general information, and how you’ve experienced it socially and emotionally [should be included].”*

*“And maybe add some quotes in there too, based on what we have said, [on] what added value [treatment] could bring to your life.”*

#### 4. Psychological Support as a Treatment-Related Health Care Need

Psychological support during treatment-related decision-making was also considered an important health care need. Participants commented on the psychological aspects of having a visible difference and its influence on decision-making, as well as the importance of emphasizing self-acceptance.

*“The psychological aspects are very important for someone who looks different…If you have another condition and look different anyways, does [the ear] even matter? And wouldn*‘*t it be better to focus on self-confidence [in these cases] instead of going for surgery?”*

In response to being asked if the PtDA could address the psychological aspects specifically, a participant noted: *“Yes, [by including], for example, a question like ‘do you expect people to treat you differently because you have an ear?’; what does someone expect to happen after having had surgery? What does someone expect will change regarding their daily lives?”*

#### 5. Factors That Influence Decision-Making

The last theme derived from the focus group discussions were the factors that influence decision-making. These factors included the extent of children's ability to participate in decision-making, as well as the influence of family dynamics and communication in this process. All participants agreed that the patient's preferences should be a driving force in decision-making, and most participating patients recalled that they had made the ultimate treatment decision for themselves, based on their preferences.

*“Everything was discussed, and not wanting [surgery] or waiting was discussed as well. We compared the advantages and disadvantages, and I made the decision myself.”*

However, there were differences in opinions on the appropriate age for involving children in decision-making. One participant strongly believed that (young) children could not be appropriately involved in decision-making:

*“I really don*‘*t think that a 9-year-old can choose. I just think that*‘*s too young. And I believe the child should be able to make the decision for themselves, and preferably they shouldn*‘*t be a child, but someone who is a bit older.”*

Parental involvement in decision-making was also discussed. For example, a participating parent recalled having influenced the ultimate treatment decision, and a participating patient explained that their mothers’ preferences had been the driving force behind decision-making:

*“[My child] chose to have a real ear. I did steer in the direction of [PPE] instead of rib cartilage, because, as a mother, I didn*‘*t like the idea of a young guy*‘*s ribcage being sawed.”*

*“My mother made the decision to have my ear operated on for me because she was afraid of me being bullied.”*

Furthermore, adoption was mentioned as a complicating factor:

*“The biggest challenge was involving [him] in [decision-making] properly, because we also have attachment and adoption-related problems. I didn*‘*t want to make the decisions for him. I did support and give a push, but I wanted to let him say what he wants.”*

Patients’ ability to participate in decision-making was also linked to knowledge of the treatment options, age and understanding. Several participants could not (accurately) recall what information they had received about the reconstructive options, where age was commonly mentioned as an associated factor:

*“I did know [that multiple surgeries were needed], but I didn*‘*t realize that [the ear] would be really different from normal during that year [in between surgeries] – and maybe that was due to [my] age, but I didn*‘*t realize that.”*

In addition to a lack of recollection, some of the participants’ comments on treatment options illustrated a (partially) inaccurate understanding of the treatment-related information:

*“I can*‘*t really remember, but I can remember something was said about a 3D ear, so maybe that was the [PPE]. I don*‘*t really know if there were other treatments than that 3D ear and the rib cartilage. As far as I can remember, it was either the rib cartilage or a 3D-ear, and you could remove that or something. I can*‘*t really remember it that well.”*

Contrastingly, both participating parents were able to explain the discussed treatment options in detail. One participating patient also noted that their father had remembered something that they themselves had not:

*“I would have liked to have known that [wire sutures are used for the surgery] because I had to have an MRI a half a year ago, and [since] my father knew that I had [wire sutures] in my ear, they had to check [if the wire is MRI safe].”*

When asked to clarify whether they thought they had received information regarding wire suture use from the HCP, the same respondent replied:

“*Yes; maybe the details get lost on you when you’re so excited to get a nice ear, but [information about] the [wire sutures] was given.”*

## Discussion

Both stakeholder groups considered realistic information about the technique, expected esthetic results, as well as possible adverse effects important to include in the PtDA. Furthermore, focus group participants considered the psychosocial effects of undergoing reconstruction as well as accounts of patient experiences important to include, whereas there was no consensus on including information on psychosocial and functional outcomes among HCPs. A recent survey of orthopedic surgeons in the USA found that the surgeons’ willingness to discuss psychosocial factors was related to their perception of resource availability (for patients and themselves), as well as their confidence in their own communication skills.^23^ Furthermore, in an interview of HCPs in cleft-related care, HCPs wanted more information and training on psychosocial factors.^24^ In the current study, patients and parents mentioned psychosocial reasons as motives to opt for reconstruction, consistent with previous reports.^25,26^ Considering these aspects, including information on psychosocial outcomes following microtia reconstruction in the PtDA seems warranted, despite the lack of consensus among HCPs. Further exploration into this lack of consensus also seems warranted.

Pediatric patients’ possible limitations in decision-making competence and parental involvement in decision-making were notable subthemes of our focus group discussions, coinciding with literature reports.^27,28^ These topics were not highlighted by many HCPs in the survey, possibly because the survey questions mainly focused on information that should be included in a PtDA. Previous studies have suggested that children become capable of making thoughtful decisions between 10 and 12 years of age.^29,30^ However, children under the age of 10 years may also be involved in decision-making in an age-appropriate way, when their developing understanding, reasoning and appreciation skills are taken into account.^31,32^ In the current study, all focus group participants agreed that pediatric patients should be involved in treatment-related decisions and that their preferences should be a driving force in the ultimate treatment choice. This is consistent with the findings of a previous qualitative study in adolescents and young adults with craniofacial microsomia, where participants advocated for including young patients in decision-making.^9^ Similarly, pediatric patients with cleft lip and palate have expressed a desire to participate in decision-making,^33,34^ especially when it comes to elective appearance-related procedures.^34^

Despite this, some parents and HCPs may prefer to opt for reconstruction before the child reaches decision-making competence due to concerns of (future) negative social interactions and their potential negative effects on the child's psychosocial functioning.^35^ Studies on ACC, PPE and OIP reconstructions have all reported benefits of treatment on psychosocial outcomes and teasing.^12,36^ Unfortunately, little research has been done on the psychosocial functioning of patients with microtia who have not undergone ear reconstruction. However, a previous prospective study reported a better psychosocial functioning in patients with microtia who had not opted for reconstruction compared to the preoperative groups of patients who had undergone reconstruction.^37^ Furthermore, there is tentative evidence to support psychological interventions in adolescents with visible differences.^38,39^ More research on the psychosocial functioning of patients with microtia who have not undergone reconstruction is clearly warranted as physicians and parents should be well informed of expected outcomes of all available options, including not undergoing reconstructive treatment, when considering treatment. Participants of the HCP survey, as well as focus group participants of the current study echoed this sentiment.

In the current study, several focus group participants could not recall the information they had received on treatment options, or recalled the information incorrectly. While it is unclear whether these inaccuracies should be contributed to an incomplete understanding due to the patients’ age, insufficient counseling or simply the elapsed time, it is notable that the recounts of both participating parents were much more accurate. This possibly suggests that counseling pediatric patients with microtia could be improved to enhance their understanding. A systematic review of SDM interventions in pediatric populations, found that PtDAs often target parents and frequently fail to involve pediatric patients themselves in the decision-making process.^40^ Considering that information tailored to families’ literacy and developmental needs has also been identified as an important facilitator of SDM,^41^ the PtDA should ideally target patients in an age-appropriate way. Patients with microtia may be very young during treatment-related decision-making, as PPE implants can be performed in children as young as 3 years.^42^ This means that information targeted to patients under the age of expected decision-making competence is also warranted. Furthermore, to facilitate discussion on possible discrepancies between pediatric patients and their parents’ preferences, preference-eliciting questions included in a PtDA should target both groups of stakeholders separately.

This study has some limitations. The small number of focus group participants who had all opted for ACC or PPE reconstruction, as well as the fact that most of them had visited the same outpatient clinic, may have influenced the generalizability of the identified health-care needs. The same themes were derived from both focus groups, suggesting that saturation was met regarding central themes for individuals who had undergone a reconstructive procedure for microtia. Nonetheless, it cannot be definitively concluded that no new themes would have been found if individuals were interviewed who did not undergo surgery. However, patients’ experiences regarding living with microtia in the current study were largely similar to previous reports in qualitative studies that also included individuals who had not opted for reconstructive surgery.^9,10^ The content of draft PtDAs should be field-tested by groups of stakeholders before definitive implementation, according to the International Patient Decision Aid Standards criteria.^43^ At this stage, developers of a PtDA should ensure that all groups of stakeholders, including individuals who opted for prosthetic reconstruction or chose not to undergo reconstructive treatment are represented, so that the content may be adjusted where necessary. Due to the mixed-methods approach, this study is also limited to general comparisons between stakeholders’ views, and a certain amount of response bias due to the provided answer options in the HCP survey cannot be ruled out.

## Conclusions

HCPs, patients and parents generally agreed on the information needed in a patient decision aid, emphasizing the importance of realistic expectation management. Patients and their parents also considered psychosocial and functional outcomes, as well as patient experiences important to include in a future decision aid. SDM in patients with microtia may be challenging due to pediatric patients’ variable ability to participate in decision-making and the influence that parents have in this process. However, patients with microtia want to be involved in treatment-related decision-making, and HCPs should encourage patient participation in an age-appropriate way. Future research may focus on evaluating and promoting SDM during counseling in this population, as well as exploring the psychosocial functioning of patients who do not opt for reconstruction.

## Implications for a PtDA for Microtia Reconstruction

- A PtDA for microtia reconstruction should target all patients with microtia and their parents and include realistic and evidence-based information on the generally available treatment options (including not undergoing reconstruction), technique-related information, expected esthetic results, possible adverse effects, and psychosocial and functional outcomes. In addition, accounts of patient experiences are considered helpful.
- A PtDA should include preference-eliciting questions for both pediatric patients and their parents.

## Supplemental Material

sj-docx-1-cpc-10.1177_10556656221146584 - Supplemental material for Stakeholders’ Views on Information Needed in a Patient Decision Aid for Microtia Reconstruction Supplemental material, sj-docx-1-cpc-10.1177_10556656221146584 for Stakeholders’ Views on Information Needed in a Patient Decision Aid for Microtia Reconstruction by E.M. Ronde, Veronique A.P. van de Lücht, N. Lachkar, Dirk T. Ubbink and Corstiaan C. Breugem in The Cleft Palate Craniofacial Journal

sj-docx-2-cpc-10.1177_10556656221146584 - Supplemental material for Stakeholders’ Views on Information Needed in a Patient Decision Aid for Microtia Reconstruction Supplemental material, sj-docx-2-cpc-10.1177_10556656221146584 for Stakeholders’ Views on Information Needed in a Patient Decision Aid for Microtia Reconstruction by E.M. Ronde, Veronique A.P. van de Lücht, N. Lachkar, Dirk T. Ubbink and Corstiaan C. Breugem in The Cleft Palate Craniofacial Journal

sj-docx-3-cpc-10.1177_10556656221146584 - Supplemental material for Stakeholders’ Views on Information Needed in a Patient Decision Aid for Microtia Reconstruction Supplemental material, sj-docx-3-cpc-10.1177_10556656221146584 for Stakeholders’ Views on Information Needed in a Patient Decision Aid for Microtia Reconstruction by E.M. Ronde, Veronique A.P. van de Lücht, N. Lachkar, Dirk T. Ubbink and Corstiaan C. Breugem in The Cleft Palate Craniofacial Journal

sj-docx-4-cpc-10.1177_10556656221146584 - Supplemental material for Stakeholders’ Views on Information Needed in a Patient Decision Aid for Microtia Reconstruction Supplemental material, sj-docx-4-cpc-10.1177_10556656221146584 for Stakeholders’ Views on Information Needed in a Patient Decision Aid for Microtia Reconstruction by E.M. Ronde, Veronique A.P. van de Lücht, N. Lachkar, Dirk T. Ubbink and Corstiaan C. Breugem in The Cleft Palate Craniofacial Journal

sj-jpg-5-cpc-10.1177_10556656221146584 - Supplemental material for Stakeholders’ Views on Information Needed in a Patient Decision Aid for Microtia Reconstruction Supplemental material, sj-jpg-5-cpc-10.1177_10556656221146584 for Stakeholders’ Views on Information Needed in a Patient Decision Aid for Microtia Reconstruction by E.M. Ronde, Veronique A.P. van de Lücht, N. Lachkar, Dirk T. Ubbink and Corstiaan C. Breugem in The Cleft Palate Craniofacial Journal

sj-docx-6-cpc-10.1177_10556656221146584 - Supplemental material for Stakeholders’ Views on Information Needed in a Patient Decision Aid for Microtia Reconstruction Supplemental material, sj-docx-6-cpc-10.1177_10556656221146584 for Stakeholders’ Views on Information Needed in a Patient Decision Aid for Microtia Reconstruction by E.M. Ronde, Veronique A.P. van de Lücht, N. Lachkar, Dirk T. Ubbink and Corstiaan C. Breugem in The Cleft Palate Craniofacial Journal

## Appendix A

**Appendix A.** Checklist for Reporting Of Survey Studies (CROSS)^a^.

Section/topic	Item	Item description	Reported on page #
Title and abstract			
Title and abstract	1a	State the word “survey” along with a commonly used term in title or abstract to introduce the study's design.	1
	1b	Provide an informative summary in the abstract, covering background, objectives, methods, findings/results, interpretation/discussion, and conclusions.	1
Introduction			
Background	2	Provide a background about the rationale of study, what has been previously done, and why this survey is needed.	2
Purpose/aim	3	Identify specific purposes, aims, goals, or objectives of the study.	2
Methods			
Study design	4	Specify the study design in the methods section with a commonly used term (eg, cross-sectional or longitudinal).	3
	5a	Describe the questionnaire (eg, number of sections, number of questions, number and names of instruments used).	3, Appendix C
Data collection methods	5b	Describe all questionnaire instruments that were used in the survey to measure particular concepts. Report target population, reported validity and reliability information, scoring/classification procedure, and reference links (if any).	3-4
	5c	Provide information on pretesting of the questionnaire, if performed (in the article or in an online supplement). Report the method of pretesting, number of times questionnaire was pre-tested, number and demographics of participants used for pretesting, and the level of similarity of demographics between pre-testing participants and sample population.	n/a
	5d	Questionnaire if possible, should be fully provided (in the article, or as appendices or as an online supplement).	Appendix C
Sample characteristics	6a	Describe the study population (ie, background, locations, eligibility criteria for participant inclusion in survey, exclusion criteria).	3-4
	6b	Describe the sampling techniques used (eg, single stage or multistage sampling, simple random sampling, stratified sampling, cluster sampling, convenience sampling). Specify the locations of sample participants whenever clustered sampling was applied.	3
	6c	Provide information on sample size, along with details of sample size calculation.	n/a
	6d	Describe how representative the sample is of the study population (or target population if possible), particularly for population-based surveys.	3
Survey administration	7a	Provide information on modes of questionnaire administration, including the type and number of contacts, the location where the survey was conducted (eg, outpatient room or by use of online tools, such as SurveyMonkey).	3
	7b	Provide information of survey's time frame, such as periods of recruitment, exposure, and follow-up days.	3
	7c	Provide information on the entry process: –>For web-based surveys, provide approaches to prevent “multiple participation” of participants.	3
Study preparation	8	Describe any preparation process before conducting the survey (eg, interviewers’ training process, advertising the survey).	n/a
Ethical considerations	9a	Provide information on ethical approval for the survey if obtained, including informed consent, institutional review board [IRB] approval, Helsinki declaration, and good clinical practice [GCP] declaration (as appropriate).	3
	9b	Provide information about survey anonymity and confidentiality and describe what mechanisms were used to protect unauthorized access.	3-4
Statistical analysis	10a	Describe statistical methods and analytical approach. Report the statistical software that was used for data analysis.	4
	10b	Report any modification of variables used in the analysis, along with reference (if available).	n/a
	10c	Report details about how missing data was handled. Include rate of missing items, missing data mechanism (ie, missing completely at random [MCAR], missing at random [MAR] or missing not at random [MNAR]) and methods used to deal with missing data (eg, multiple imputation).	4
	10d	State how non-response error was addressed.	n/a
	10e	For longitudinal surveys, state how loss to follow-up was addressed.	n/a
	10f	Indicate whether any methods such as weighting of items or propensity scores have been used to adjust for non-representativeness of the sample.	n/a
	10g	Describe any sensitivity analysis conducted.	n/a
Results			
Respondent characteristics	11a	Report numbers of individuals at each stage of the study. Consider using a flow diagram, if possible.	5
	11b	Provide reasons for nonparticipation at each stage, if possible.	n/a
	11c	Report response rate, present the definition of response rate or the formula used to calculate response rate.	5
	11d	Provide information to define how unique visitors are determined. Report number of unique visitors along with relevant proportions (eg, view proportion, participation proportion, completion proportion).	Table 1,table 2
Descriptive results	12	Provide characteristics of study participants, as well as information on potential confounders and assessed outcomes.	5, table 1
Main findings	13a	Give unadjusted estimates and, if applicable, confounder-adjusted estimates along with 95% confidence intervals and p-values.	5
	13b	For multivariable analysis, provide information on the model building process, model fit statistics, and model assumptions (as appropriate).	n/a
	13c	Provide details about any sensitivity analysis performed. If there are considerable amount of missing data, report sensitivity analyses comparing the results of complete cases with that of the imputed dataset (if possible).	n/a
Discussion			
Limitations	14	Discuss the limitations of the study, considering sources of potential biases and imprecisions, such as non-representativeness of sample, study design, important uncontrolled confounders.	13
Interpretations	15	Give a cautious overall interpretation of results, based on potential biases and imprecisions and suggest areas for future research.	11-14
Generalizability	16	Discuss the external validity of the results.	11-13
Other sections			
Role of funding source	17	State whether any funding organization has had any roles in the survey's design, implementation, and analysis.	20
Conflict of interest	18	Declare any potential conflict of interest.	20
Acknowledgements	19	Provide names of organizations/persons that are acknowledged along with their contribution to the research.	n/a

^a^ Developed from: Sharma A, Minh Duc NT, Luu Lam Thang T, et al. A Consensus-Based Checklist for Reporting of Survey Studies (CROSS). J Gen Intern Med. Apr 22 2021; doi:10.1007/s11606-021-06737-1.

## Appendix B

**Appendix B.** Consolidated criteria for reporting qualitative studies (COREQ)^a^.

Item No.	Item	Guide questions/description	Response	Reported on page #
Domain 1: Research team and reflexivity				
Personal Characteristics				
1.	Interviewer/facilitator	Which author/s conducted the interview or focus group?	EMR, VAPL and DTU	n/a
2.	Credentials	What were the researcher's credentials? Eg, *PhD, MD*	EMR and VAPL: MD; DTU: MD, PhD	n/a
3.	Occupation	What was their occupation at the time of the study?	EMR: PhD candidate, VAPL: student researcher DTU: full professor	n/a
4.	Gender	Was the researcher male or female?	Female (EMR, VAPL)) and male (DTU)	n/a
5.	Experience and training	What experience or training did the researcher have?	DTU: professor of evidence-based medicine and shared decision-making, has experience in moderating focus group discussions. EMR and VAPL familiarized themselves with conducting focus groups through available literature and discussion with experiences peers.	n/a
Relationship with participants				
6.	Relationship established	Was a relationship established prior to study commencement?	Relationship was established at inclusion for this study. No contact was initiated by the researchers prior to this study.	n/a
7.	Participant knowledge of the interviewer	What did the participants know about the researcher? eg, *personal goals, reasons for doing the research*	Reasons for doing research. Personal details were not discussed.	n/a
8.	Interviewer characteristics	What characteristics were reported about the interviewer/facilitator? eg, *Bias, assumptions, reasons and interests in the research topic*	An independent moderator was selected to avoid bias, assumptions, reasons and interests in the research topic as much as possible.	n/a
Domain 2: study design				
Theoretical framework				
9.	Methodological orientation and Theory	What methodological orientation was stated to underpin the study? eg, *grounded theory, discourse analysis, ethnography, phenomenology, content analysis*	Phenomenology	n/a
Participant selection				
10.	Sampling	How were participants selected? eg, *purposive, convenience, consecutive, snowball*	Purposive (within Amsterdam UMC) and voluntary (patient organization)	4
11.	Method of approach	How were participants approached? eg, *face-to-face, telephone, mail, email*	Initially face-to-face (patient organization Laposa members’ meeting: online video conference) and email (Amsterdam UMC). Subsequently by telephone (consent and participation details).	n/a
12.	Sample size	How many participants were in the study?	8 (focus groups)	6
13.	Nonparticipation	How many people refused to participate or dropped out? Reasons?	1 (clashing activities)	6
Setting				
14.	Setting of data collection	Where was the data collected? eg, *home, clinic, workplace*	Online	4
15.	Presence of non-participants	Was anyone else present besides the participants and researchers?	No	n/a
16.	Description of sample	What are the important characteristics of the sample? eg, *demographic data, date*	See manuscript	6, table 3
Data collection				
17.	Interview guide	Were questions, prompts, guides provided by the authors? Was it pilot tested?	Yes, not tested.	Appendix D
18.	Repeat interviews	Were repeat interviews carried out? If yes, how many?	No	n/a
19.	Audio/visual recording	Did the research use audio or visual recording to collect the data?	Yes	4
20.	Field notes	Were field notes made during and/or after the interview or focus group?	Yes	4
21.	Duration	What was the duration of the interviews or focus group?	90 min × 2	n/a
22.	Data saturation	Was data saturation discussed?	Yes	14
23.	Transcripts returned	Were transcripts returned to participants for comment and/or correction?	No	n/a
Domain 3: analysis and findings				
Data analysis				
24.	Number of data coders	How many data coders coded the data?	2	4-5
25.	Description of the coding tree	Did authors provide a description of the coding tree?	Yes	Figure 2
26.	Derivation of themes	Were themes identified in advance or derived from the data?	Yes	5
27.	Software	What software, if applicable, was used to manage the data?	Microsoft word, SPSS	4
28.	Participant checking	Did participants provide feedback on the findings?	No	n/a
Reporting				
29.	Quotations presented	Were participant quotations presented to illustrate the themes / findings? Was each quotation identified? eg, *participant number*	Yes, however participants are not identified to ensure anonymity.	6-12
30.	Data and findings consistent	Was there consistency between the data presented and the findings?	Yes	6-14, Figure 2
31.	Clarity of major themes	Were major themes clearly presented in the findings?	Yes	6-14, Figure 2
32.	Clarity of minor themes	Is there a description of diverse cases or discussion of minor themes?	Yes	6-14, Figure 2

^a^ Developed from: Tong A, Sainsbury P, Craig J. Consolidated criteria for reporting qualitative research (COREQ): a 32-item checklist for interviews and focus groups. Int J Qual Health Care. 2007;19(6):349-357.

## Appendix C. Clinician survey questions

*Participant details*

## Appendix D. Focus group guide and supporting questions

*Introduction/general information:*

- Introduce yourself and your relation to microtia (patient/parent)
- Has your ear been treated/operated?
- Were you involved in decision making?
- Were multiple treatment options discussed?

*Questions on received information:*

- What do you think of the information you received about the ear reconstruction options?
  ○ Which information did you think was good and why?
  ○ Which information did you think was poor and why?
  ○ Which information would you have liked to have received and why?

*Questions about decision-making:*

- Why did you opt to undergo/not undergo surgery?
- Why did you opt for rib cartilage/medpor/external prosthesis?
  ○ Which considerations were important for you during decision-making?
    ▪ Which considerations were the most important?
  ○ Which considerations do you think physicians may not consider during counseling that are important for patients?
- How would you advise others (considering treatment) to contemplate their options?

*Decision-aid rough prototype:*

- What do you think of the first rough prototype of the decision aid (what is good, what can be improved upon)?

*Wrap-up:*

- Final comments (if any).
