# Supplementary material for: Stakeholders’ Views on Information Needed in a Patient Decision Aid for Microtia Reconstruction
Source: Cleft Palate Craniofac J. 2023 Jan 5;61(5):854–69. doi: 10.1177/10556656221146584 (PMC10981206; doi:10.1177/10556656221146584)
Supplement: sj-docx-2-cpc-10.1177_10556656221146584 - Supplemental material for Stakeholders’ Views on Information Needed in a Patient Decision Aid for Microtia Reconstruction [file sj-docx-2-cpc-10.1177_10556656221146584.docx]

**Appendix B:** Consolidated criteria for reporting qualitative studies (COREQ) *

| **Item No.** | **Item** | **Guide questions/description** | **Response** | **Reported on page #** |
| --- | --- | --- | --- | --- |
| **Domain 1: Research team and reflexivity** | | | |  |
| Personal Characteristics | | | |  |
| 1. | Interviewer/facilitator | Which author/s conducted the interview or focus group? | First, second, second to last [redacted initials] | n/a |
| 2. | Credentials | What were the researcher's credentials? *E.g. PhD, MD* | First and second: MD; second to last: MD, PhD [initials redacted] | n/a |
| 3. | Occupation | What was their occupation at the time of the study? | First: PhD candidate, Second: student researcher  Second to last: full professor [initials redacted] | n/a |
| 4. | Gender | Was the researcher male or female? | Female (first, second) and male (second to last) [initials redacted] | n/a |
| 5. | Experience and training | What experience or training did the researcher have? | Second to last: professor of evidence-based medicine and shared decision-making, has experience in moderating focus group discussions. First and second familiarized themselves with conducting focus groups through available literature and discussion with experiences peers. [initials redacted] | n/a |
| Relationship with participants | | | |  |
| 6. | Relationship established | Was a relationship established prior to study commencement? | Relationship was established at inclusion for this study. No contact was initiated by the researchers prior to this study. | n/a |
| 7. | Participant knowledge of the interviewer | What did the participants know about the researcher? e*.g. personal goals, reasons for doing the research* | Reasons for doing research. Personal details were not discussed. | n/a |
| 8. | Interviewer characteristics | What characteristics were reported about the interviewer/facilitator? e.g. *Bias, assumptions, reasons and interests in the research topic* | An independent moderator was selected to avoid bias, assumptions, reasons and interests in the research topic as much as possible. | n/a |
| **Domain 2: study design** | | | |  |
| Theoretical framework | | | |  |
| 9. | Methodological orientation and Theory | What methodological orientation was stated to underpin the study? *e.g. grounded theory, discourse analysis, ethnography, phenomenology, content analysis* | Phenomenology | n/a |
| Participant selection | | | |  |
| 10. | Sampling | How were participants selected? *e.g. purposive, convenience, consecutive, snowball* | Purposive (within hospital [name redacted]) and voluntary (patient organization) | 4 |
| 11. | Method of approach | How were participants approached? e*.g. face-to-face, telephone, mail, email* | Initially face-to-face (patient organization [name redacted] members’ meeting: online video conference) and email ([hospital name redacted]). Subsequently by telephone (consent and participation details). | n/a |
| 12. | Sample size | How many participants were in the study? | 8 (focus groups) | 6 |
| 13. | Non-participation | How many people refused to participate or dropped out? Reasons? | 1 (clashing activities) | 6 |
| Setting |  |  |  |  |
| 14. | Setting of data collection | Where was the data collected? e*.g. home, clinic, workplace* | Online | 4 |
| 15. | Presence of non-participants | Was anyone else present besides the participants and researchers? | No | n/a |
| 16. | Description of sample | What are the important characteristics of the sample? *e.g. demographic data, date* | See manuscript | 6, table 3 |
| Data collection | | |  |  |
| 17. | Interview guide | Were questions, prompts, guides provided by the authors? Was it pilot tested? | Yes, not tested. | Appendix D |
| 18. | Repeat interviews | Were repeat interviews carried out? If yes, how many? | No | n/a |
| 19. | Audio/visual recording | Did the research use audio or visual recording to collect the data? | Yes | 4 |
| 20. | Field notes | Were field notes made during and/or after the interview or focus group? | Yes | 4 |
| 21. | Duration | What was the duration of the interviews or focus group? | 90 min x 2 | n/a |
| 22. | Data saturation | Was data saturation discussed? | Yes | 14 |
| 23. | Transcripts returned | Were transcripts returned to participants for comment and/or correction? | No | n/a |
| **Domain 3: analysis and findings** | | | |  |
| Data analysis | | | |  |
| 24. | Number of data coders | How many data coders coded the data? | 2 | 4-5 |
| 25. | Description of the coding tree | Did authors provide a description of the coding tree? | Yes | Figure 2 |
| 26. | Derivation of themes | Were themes identified in advance or derived from the data? | Yes | 5 |
| 27. | Software | What software, if applicable, was used to manage the data? | Microsoft word, SPSS | 4 |
| 28. | Participant checking | Did participants provide feedback on the findings? | No | n/a |
| Reporting |  |  |  |  |
| 29. | Quotations presented | Were participant quotations presented to illustrate the themes / findings? Was each quotation identified? e*.g. participant number* | Yes, however participants are not identified to ensure anonymity. | 6-12 |
| 30. | Data and findings consistent | Was there consistency between the data presented and the findings? | Yes | 6-14, figure 2 |
| 31. | Clarity of major themes | Were major themes clearly presented in the findings? | Yes | 6-14, figure 2 |
| 32. | Clarity of minor themes | Is there a description of diverse cases or discussion of minor themes? | Yes | 6-14, figure 2 |

* Developed from: Tong A, Sainsbury P, Craig J. Consolidated criteria for reporting qualitative research (COREQ): a 32-item checklist for interviews and focus groups. Int J Qual Health Care. 2007;19(6):349-357.
