# Supplementary figures and images for: Stakeholders’ Views on Information Needed in a Patient Decision Aid for Microtia Reconstruction
Source: Cleft Palate Craniofac J. 2023 Jan 5;61(5):854–69. doi: 10.1177/10556656221146584 (PMC10981206; doi:10.1177/10556656221146584)

**Appendix C:** Clinician survey questions

*Participant details*


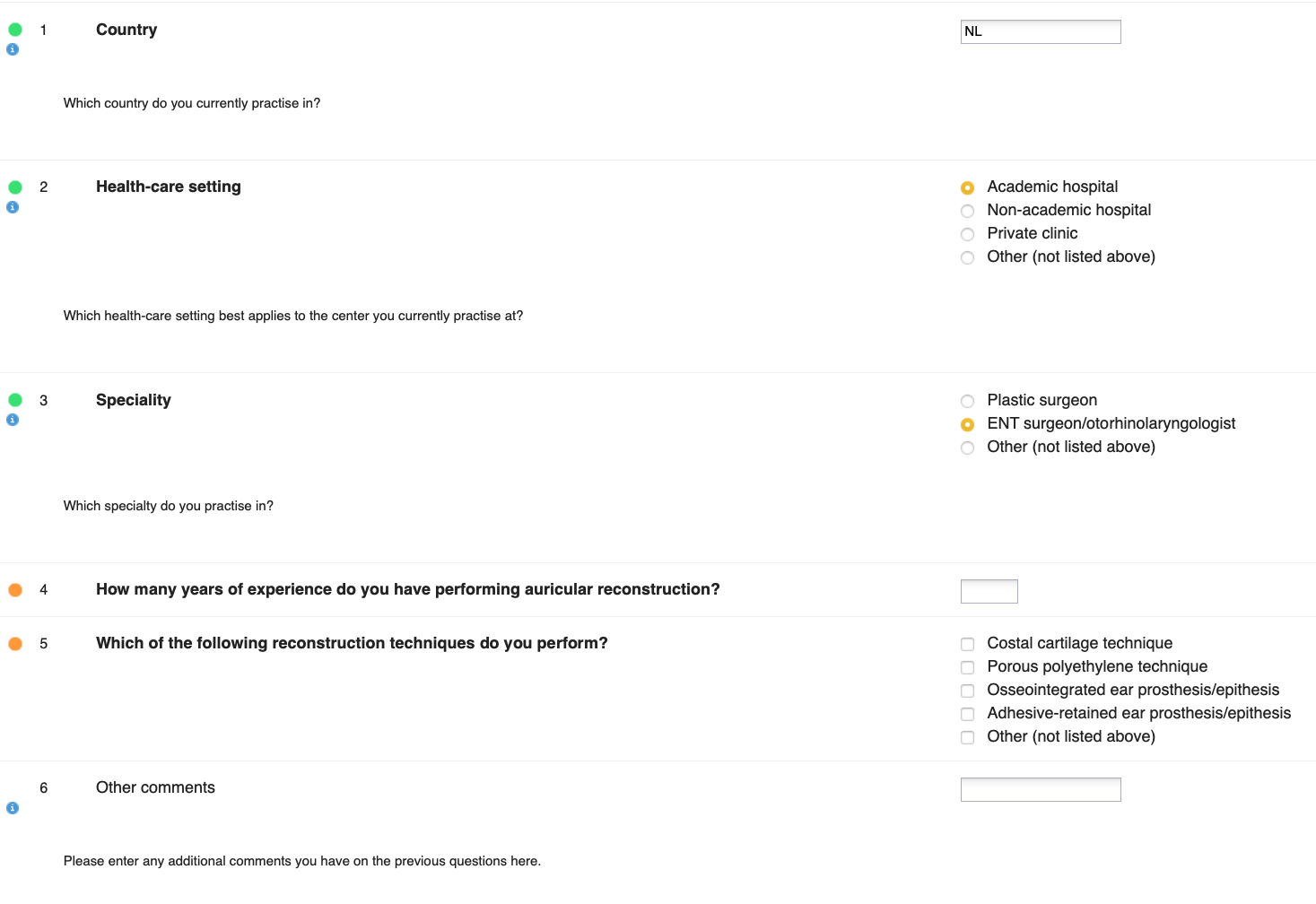


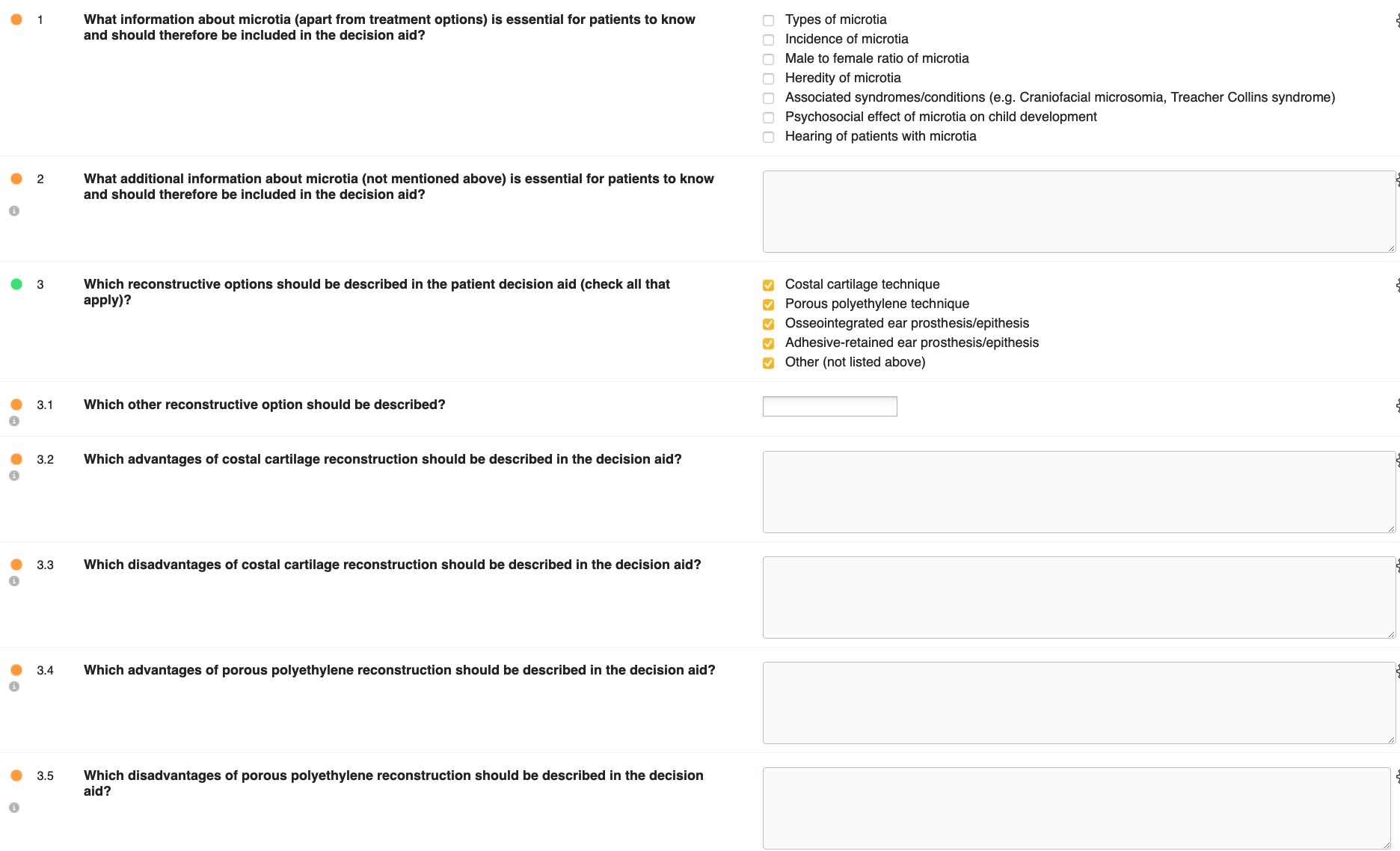
*Questions on PtDA content*


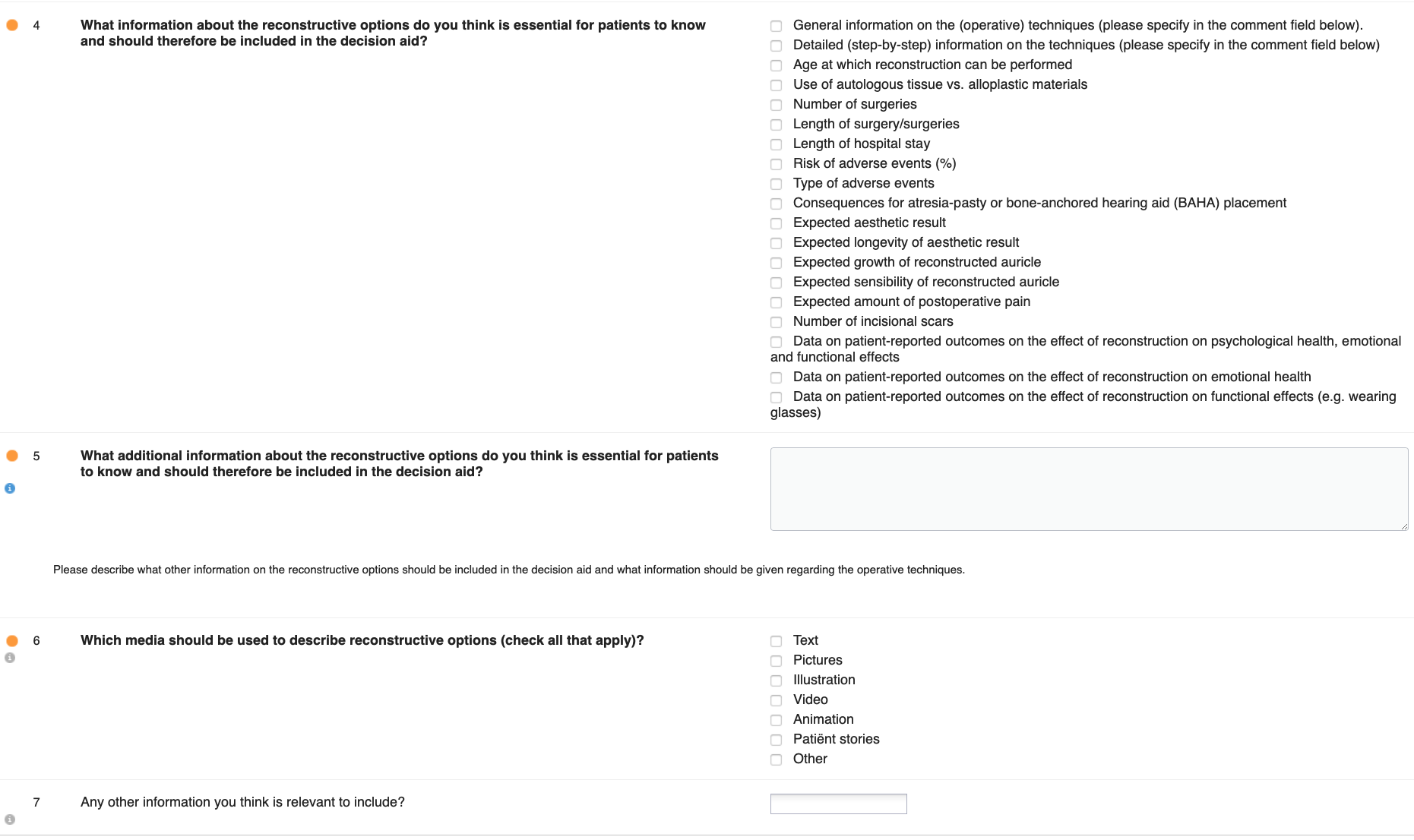

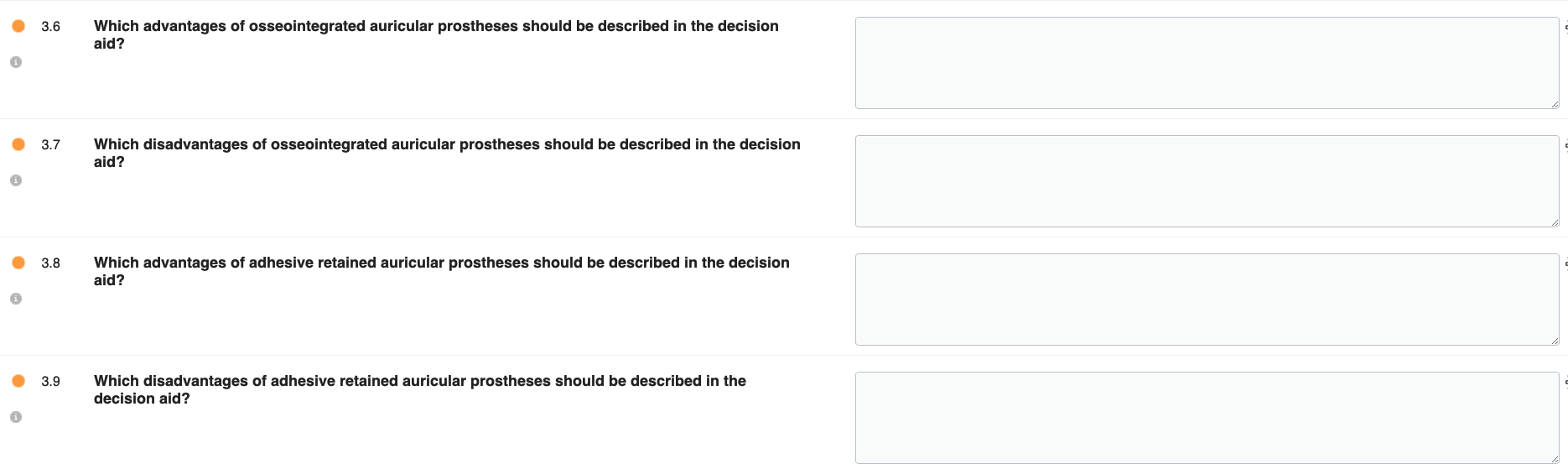


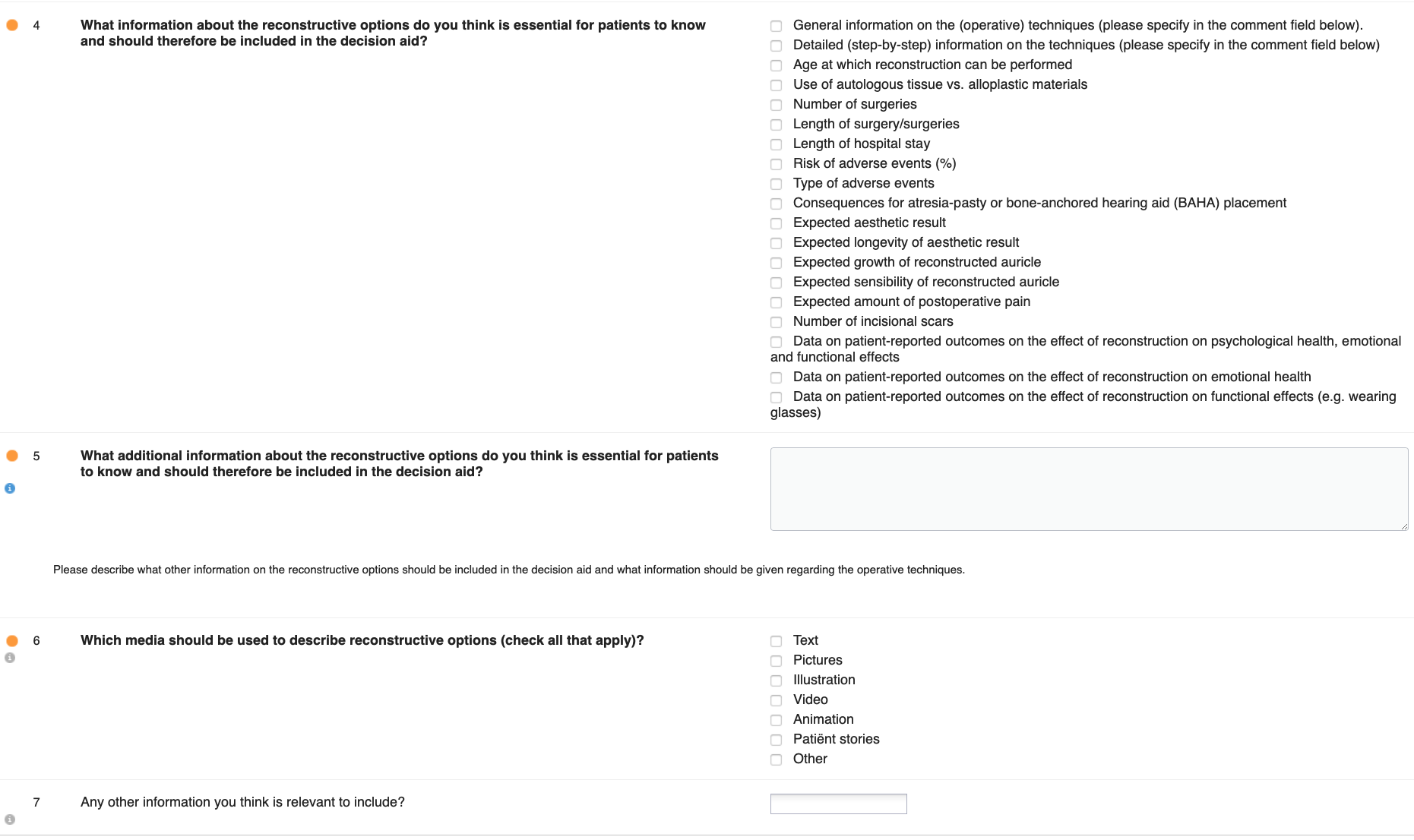

Supplement: sj-docx-3-cpc-10.1177_10556656221146584 - Supplemental material for Stakeholders’ Views on Information Needed in a Patient Decision Aid for Microtia Reconstruction [file sj-docx-3-cpc-10.1177_10556656221146584.docx]

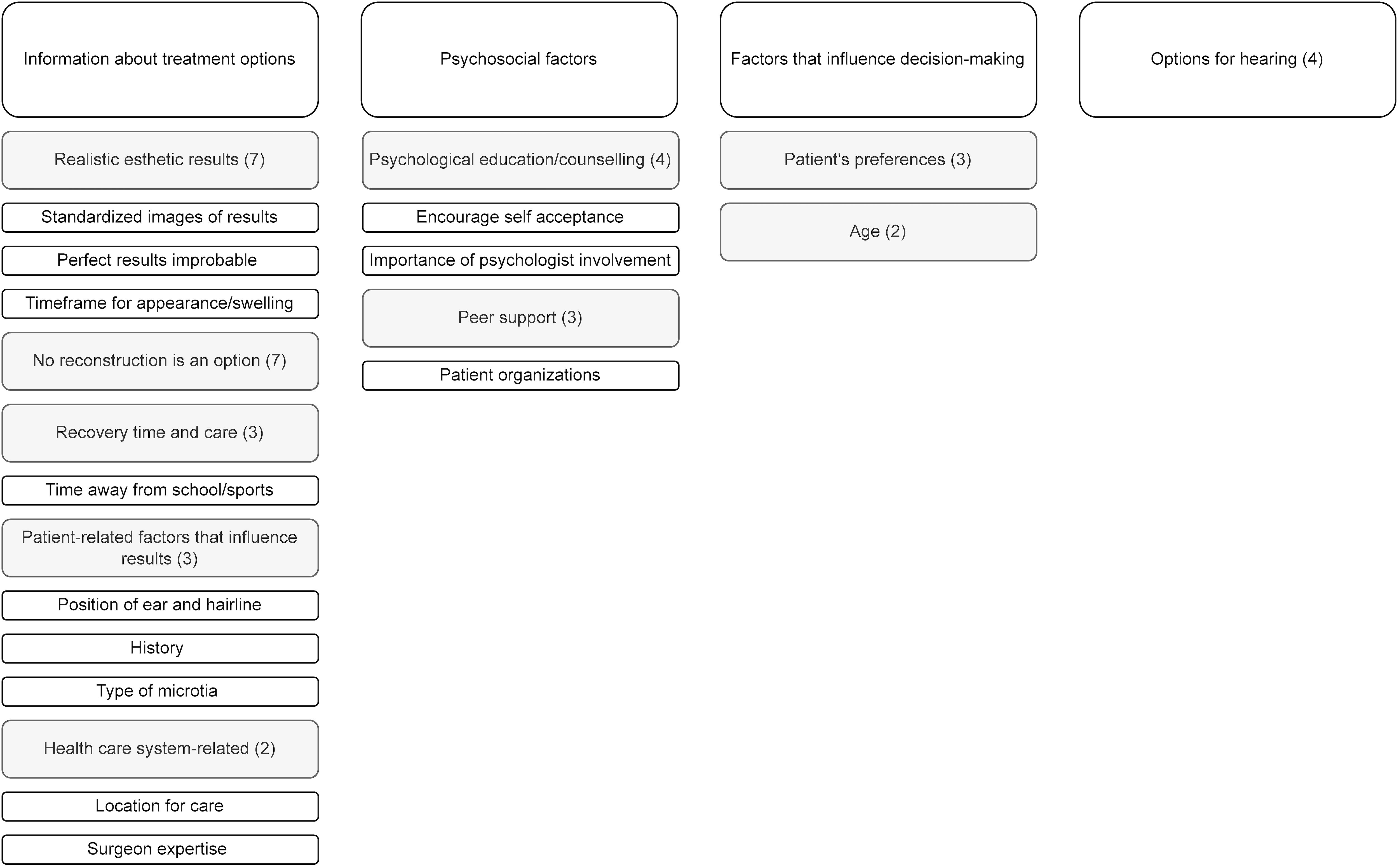

Supplement: sj-jpg-5-cpc-10.1177_10556656221146584 - Supplemental material for Stakeholders’ Views on Information Needed in a Patient Decision Aid for Microtia Reconstruction [file sj-jpg-5-cpc-10.1177_10556656221146584.jpg]
