# Supplementary material for: Stakeholders’ Views on Information Needed in a Patient Decision Aid for Microtia Reconstruction
Source: Cleft Palate Craniofac J. 2023 Jan 5;61(5):854–69. doi: 10.1177/10556656221146584 (PMC10981206; doi:10.1177/10556656221146584)
Supplement: sj-docx-4-cpc-10.1177_10556656221146584 - Supplemental material for Stakeholders’ Views on Information Needed in a Patient Decision Aid for Microtia Reconstruction [file sj-docx-4-cpc-10.1177_10556656221146584.docx]

**Appendix D:** Focus group guide and supporting questions

Introduction/general information:

- Introduce yourself and your relation to microtia (patient/parent)
- Has your ear been treated/operated?
- Were you involved in decision making?
- Were multiple treatment options discussed?

Questions on received information:

- What do you think of the information you received about the ear reconstruction options?
  - Which information did you think was good and why?
  - Which information did you think was poor and why?
  - Which information would you have liked to have received and why?

Questions about decision-making:

- Why did you opt to undergo/not undergo surgery?
- Why did you opt for rib cartilage/medpor/external prosthesis?
  - Which considerations were important for you during decision-making?
    - Which considerations were the most important?
  - Which considerations do you think physicians may not consider during counselling that are important for patients?
- How would you advise others (considering treatment) to contemplate their options?

Decision-aid rough prototype:

- What do you think of the first rough prototype of the decision aid (what is good, what can be improved upon)?

Wrap-up:

- Final comments (if any).
