# Supplementary material for: Stakeholders’ Views on Information Needed in a Patient Decision Aid for Microtia Reconstruction
Source: Cleft Palate Craniofac J. 2023 Jan 5;61(5):854–69. doi: 10.1177/10556656221146584 (PMC10981206; doi:10.1177/10556656221146584)
Supplement: sj-docx-6-cpc-10.1177_10556656221146584 - Supplemental material for Stakeholders’ Views on Information Needed in a Patient Decision Aid for Microtia Reconstruction [file sj-docx-6-cpc-10.1177_10556656221146584.docx]

Supplementary figure 1. Code tree of themes derived from qualitative survey responses by health care providers.
